# Effect of Dry Needling Treatment on Tibial Musculature in Combination with Neurorehabilitation Treatment in Stroke Patients: Randomized Clinical Study

**DOI:** 10.3390/ijerph191912302

**Published:** 2022-09-28

**Authors:** Zacarías Sánchez Milá, Jorge Velázquez Saornil, Angélica Campón Chekroun, José Manuel Barragán Casas, Raúl Frutos Llanes, Arantxa Castrillo Calvillo, Cristina López Pascua, David Rodríguez Sanz

**Affiliations:** 1 Area of Physiotherapy, Catholic University of Ávila, 05005 Ávila, Spain; 2 Centro Lescer, 28050 Madrid, Spain; 3 Faculty of Nursing, Physiotherapy and Podiatry, Universidad Complutense de Madrid, 28040 Madrid, Spain

**Keywords:** stroke, ultrasound-guided dry needling, spasticity, neurorehabilitation

## Abstract

(1) Background: Introducing ultrasound-guided dry needling to neurorehabilitation treatments increases the beneficial effects of therapy. The aim of this study was to compare the effects of including an ultrasound-guided dry needling session in neurorehabilitation treatment on spasticity and gait–balance quality versus neurorehabilitation treatment in subjects who had suffered a stroke. (2) Methods: A single-blind, randomized clinical trial was conducted. Thirty-six patients who had suffered a stroke in the right middle cerebral artery signed the informed consent for participation in the study. Twenty patients finally participated and were randomly assigned to the control group (neurorehabilitation treatment) or experimental group (neurorehabilitation treatment plus ultrasound-guided dry needling). Pre-treatment and post-treatment data were collected on the same day. The experimental group (n = 10) first underwent an ultrasound-guided dry needling intervention on the tibialis anterior and tibialis posterior musculature, followed by neurorehabilitation treatment; the control group (n = 10) underwent their corresponding neurorehabilitation without the invasive technique. Pre-treatment and post-treatment measurements were taken on the same day, assessing the quality of balance–gait using the “Up and Go” test and the degree of spasticity using the Modified Modified Ashworth Scale. (3) Results: The patients who received neurorehabilitation treatment plus ultrasound-guided dry needling showed a greater decrease in spasticity in the tibial musculature after the neurorehabilitation treatment session (*p* < 0.001), improving balance and gait (*p* < 0.001). (4) Conclusions: An ultrasound-guided dry needling session combined with neurorehabilitation treatment reduced spasticity and improved balance and gait in stroke patients.

## 1. Introduction

Worldwide, the incidence of people who suffer a stroke each year, according to the World Health Organization (WHO), is approximately 15 million; of these, 5.5 million people die, and the rest of the people who survive are permanently disabled [1]. According to the World Stroke Organisation (WSO), there are 80 million people with various disabilities whose quality of life is attenuated along with the socio-economic impact of these disabilities [2].

When a neurological lesion occurs, it is known that two types of hypertonia can occur: rigidity, in the case of the involvement of the extrapyramidal pathway, at the level of the basal ganglia; or spasticity, which is produced by a lesion of the pyramidal pathway [3]. Spasticity affects approximately more than 35% of people with stroke (ischemic and haemorrhagic) [4], progresses to chronicity and is capable of causing soft tissue changes; this results in muscle fibrosis, an abnormal chronic enlargement of muscle fibres and extracellular matrix, as it occurs in the main muscle protein, collagen, dominated by the type I and type III isoforms of skeletal muscle, and leads to the reprogramming of resident fibroblasts, fibroadipogenic progenitors, pericytes and mesoangioblasts, causing muscle retractions, osteoarticular deformities and thus pain [5]. Changes in the spastic musculature of patients with upper motor neuron involvement include altered fibre type and fibre size distribution; the proliferation of extracellular matrix material; increased stiffness of spastic muscle cells and, to a lesser extent, spastic muscle tissue; and inferior mechanical properties of extracellular material in spastic musculature compared to normal muscle [6].

Dry needling (DN) is an invasive technique that involves the use of needles in the subcutaneous tissues and muscle for the treatment of myofascial trigger points (MTrPs); unlike other techniques, DN does not introduce any substance into the body [7], with a mechanism of action on the muscle tension bands, causing the separation between actin and myosin present in the contracted sarcomeres [8]. It can also destroy, via the mechanical stimulation of the needle, the motor plate by modifying the acetylcholine and choline esterase receptors, as it occurs in muscle regeneration, promoting tissue remodelling; this is possible because the common diameter of the needles used is between 160 and 450 µm, while the diameter of the normal adult myocyte is 20–60 µm, so the application of DN can induce tissue damage in the muscle fibre, the motor plate and axon [9,10]. All these mechanisms of action have a local spasm response, which is produced when the puncture needle is introduced in the MTrP, causing an increase in blood flow, and this results in a decrease in algaeogenic compounds in the area [11].

Neurorehabilitation protocols are based on different types of neurorehabilitation approaches (Bobath Concept, Proprioceptive Neuromuscular Facilitation, Constraint Induced Movement Therapy, etc.), and different therapeutic tools (neuromuscular taping, orthoses, robotic aids, dry needling, etc.) can be used for the efficacy of individual programs for neurological patients; among them, dry needling (focused on the lower limb), together with a neurorehabilitation programme, was shown to obtain a moderate validity in the reduction in spasticity [12].

For this reason, our research team set out to analyse the changes in spasticity, balance and gait in the short term (one day of treatment) in stroke patients via an ultrasound-guided DN intervention on the tibial musculature together with neurorehabilitation treatment versus neurorehabilitation treatment without receiving invasive techniques.

## 2. Materials and Methods

A randomized blinded controlled experimental study was carried out. The protocol was registered on the clinicaltrials.gov website and was admitted and assigned the following identifier number: NCT05230849.

The authors declare that this intervention was performed with the prior informed consent of the patients. This is a human subjects research study approved by the ethics committee of Hospital Sonsoles of Ávila (Spain) with number GASAV/2022/1 and was conducted according to the Declaration of Helsinki biomedical guidelines.

### 2.1. Participants

Participants were recruited at the Lescer clinic located in Madrid (Spain), following these inclusion criteria: (1) clinical history of a stroke due to ischaemia in the right middle cerebral artery in the subacute phase with six months of evolution; (2) walking independence; (3) ankle instability (equinus, varus, equinovarus); (4) age between 18 and 80 years. Participants were excluded due to these exclusion criteria: (1) repeat stroke; (2) being treated in the lower limb musculature with botulinum toxin; (3) possessing severe cognitive deficits; (4) belonephobia (needle phobia); (5) allergy to dry needling needle material.

### 2.2. Group Assignment

Participants were informed of the study and agreed to participate by signing the informed consent form. They were randomly assigned to two groups. The intervention group received ultrasound-guided dry needling in the tibial musculature followed by neurorehabilitation therapy (NRT); on the other hand, the control group received NRT without invasive techniques.

For the division of the groups, participants picked up a piece of paper from an opaque box, on which a number could be seen: 1 corresponded to the intervention group or 2 corresponded to the control group.

Minutes before the treatment (lasting one day), the participants were assessed (lift and gait test and modified Ashworth scale) by their physiotherapists (the therapist was blind). The DN group underwent the invasive intervention together with ultrasound guidance, and both groups were treated with NRT by their physiotherapists.

### 2.3. Protocol Interventions

#### 2.3.1. Gait and Balance Evaluation

The “Up and Go” test (TUG) was developed in 1991 from the “Get Up and Go” test. It is used primarily in the elderly population to assess the risk of falls, as well as to assess gait and balance. It can also be used in patients who have suffered a stroke or present with vertigo or in people with arthritis. To perform the test, the patient has to get up from a chair, walk three meters and return to the starting point. A first trial test is performed, during which the patient can go at the speed he/she wishes and use technical aids such as a cane or a walker if necessary, with the results expressed in seconds [13,14].

#### 2.3.2. Modified Modified Ashworth Scale

It is important to measure spasticity in order to evaluate the impact of treatments performed on patients, and one of the most widely used scales to assess spasticity in the lower limbs is the Modified Modified Ashworth Scale (MMAS) [15,16,17].

The assessment of the patients was carried out in the supine position with the physiotherapist homolateral to the affected side; a stabilising shot was given at the proximal third of the tibioperoneal joint (to avoid knee flexion compensation), and another mobilising shot was given at the Chopart and Lisfranc joints. The action was to perform five fast movements in each direction, i.e., plantar flexion–supination and dorsal flexion with slight supination, thus assessing the tibialis posterior and tibialis anterior. The result of the test was marked according to the level of resistance of the musculature to the fast movements, valued from 0 to 4, where 0 = no increase in muscle tone; 1 = slight increase in muscle tone, manifested as a catch and release or as minimal resistance at the end of the range of motion when the affected part(s) was moved in flexion/extension; 2 = marked increase in muscle tone, manifested as a catch in the middle range and resistance throughout the remainder of the range of motion, with the affected part(s) being easily moved; 3 = considerable increase in muscle tone, passive movement difficult; and 4 = the affected part(s) showed to be rigid in flexion or extension.

#### 2.3.3. Neurorehabilitation Therapy

All participants received NRT, which lasted 60 min on a single day, and each person was assessed individually and in detail. In all cases, the lack of postural control in static and dynamic standing due to the misalignment of the most affected foot in inversion was identified as the main problem, and a common treatment plan was devised: seated lying down position to align the hips, to activate the central stabilisers and to mobilise the affected foot to improve foot–floor interaction; prone standing to activate the trunk extensors against gravity, to gain latissimus dorsi length by activating the shoulder girdle and to perform eccentric work of the most affected lower limb; in the standing position, the loading of the most affected lower limb, with heel contact to facilitate selective extension in the affected leg and eccentric work on the posterior chain of the most affected side with stability on the less affected side, simulating swing phases and correcting foot support; finally, gait facilitation during the last five minutes of the treatment session [18,19,20].

### 2.4. Ultrasound-Guided Dry Needling

**Figure 1:**
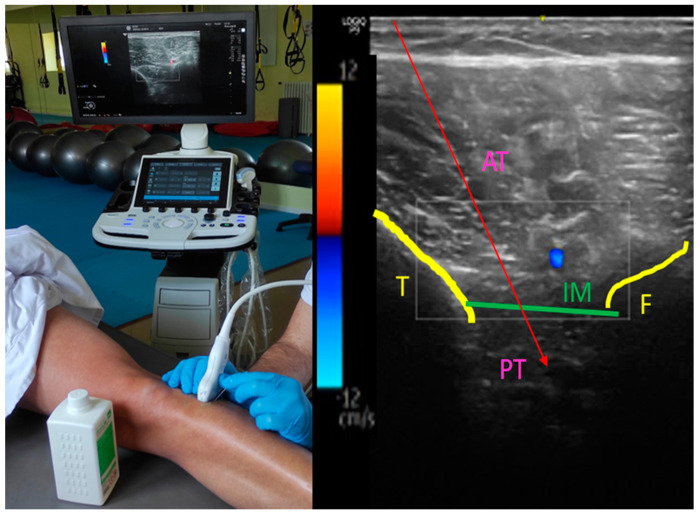
Ultrasound−guided dry needling. Upper third of the leg; AT, followed by the interosseous membrane (IM) as a hyperechoic band between the tibia (T) and fibula (F), sparing the neurovascular component (anterior tibial artery and vein, and deep peroneal nerve), to finally visualise PT.

The experimental group also received DN therapy with disposable 0.30 × 60 mm stainless steel Agupunt^®^ needles guided using ultrasound with a 3–12 MHz probe, with programme-determined musculoskeletal and deep vision, in a transversal probe position, with the subjects being positioned in supine decubitus in the posterior tibialis (PT) as plantarflexor and the anterior tibialis (AT) as dorsiflexor (Figure 1). Both were coactivated for subjects presenting with a varus foot deformity, which leads to losing hindfoot stability and gait, thus decreasing the subjects’ quality of life [21,22].

In order to approach the AT and PT together, first, the skin was cleaned with 250 mL of Chlorhexidine 2% Aqueous Spray; then, the ultrasound-guided needle was introduced into the AT to perform DN therapy for one minute, with slow entry and exit movements (to avoid the withdrawal reflex due to pain) and angling in different directions to achieve local spasm response; we continued performing the same technique in the PT, avoiding the neurovascular bundle. When the dry needling technique was completed, the stretching of the musculature was performed to continue with NRT. These techniques were carried out by the same therapist.

### 2.5. Statistical Study

The data were analysed using SPSS version 28.0. The descriptive results of continuous data were expressed as means and standard deviation, while nominal data were described as frequencies and percentages. The normal distribution of the variables was tested using the Shapiro–Wilk test, resulting in *p* > 0.05 for the TUG test; we proceeded to analyse the results before and after with a 2 *×* 2 within-subjects ANOVA, considering the homogeneity of variances by means of the Levene test. The results were *p* < 0.05 for the MMAS clinical test; we proceeded to analyse the results before and after with the Mann–Whitney U test, with *p* < 0.05 being statistically significant

## 3. Results

**Figure 2:**
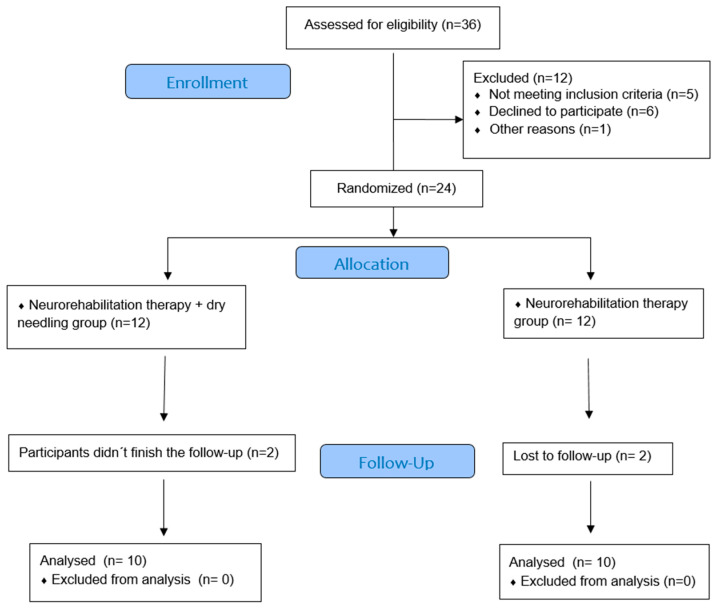
Consort 2010 flow diagram.

Thirty-six patients who had suffered a stroke and were undergoing a rehabilitation protocol at the Lescer clinic (Madrid, Spain) voluntarily participated in the study. Finally, twenty patients (49.25 ± 8.27 years; men 65%, women 35%) met the eligibility criteria, agreed to participate and were randomised into the neurorehabilitation therapy group (n = 10) or the neurorehabilitation therapy plus dry needling group (n = 10). Reasons for ineligibility and loss of subjects are given in the flow chart (Figure 2). Demographic and clinical characteristics of the participants by groups are depicted in Table 1.

### 3.1. Changes in the Spasticity

The non-parametric Mann–Whitney U test revealed a significant reduction in MMAS at the PT (*p* = <0.001) and AT (*p* = <0.001) after intervention in the neurorehabilitation therapy plus dry needling group (Table 2).

### 3.2. Changes in Gait Coordination

To detect changes before and after the intervention and between-group differences in the TUG test, a 2 × 2 within-subjects ANOVA was applied. Significant improvements were obtained in the dry needling group (F = 22.71, *p* < 0.001).

## 4. Discussion

The results of the study indicated that those patients who had suffered a stroke and who received DN therapy on the spastic musculature of the lower limb with moderate validation [23], specifically, on the AT and PT musculature, combined with a treatment session according to NRT obtained a greater decrease in spasticity, measured using the modified Ashworth scale, as it is one of the most widely used scales at present [24]. Rabita et al. [25] compared the results obtained using the traditional assessment of the Ashworth scale and the manual passive mobilization of ankle dorsiflexion with isokinetic movement, determining that the speeds at which the test was performed were different and that using manual execution, it was easier to evoke the stretch reflex, which could yield subjective values depending on the evaluator. In our study, it was the same evaluator, blinded to the assigned treatment group, who evaluated all patients under the clinical criterium established by Biering Sorensen et al. [26] of one second to complete the joint pathway, so that all patients were evaluated according to similar parameters, thus gaining greater static and dynamic stability in the joint treatment session.

Currently, stroke among older adults is considered a major generator of disability, which not only implies a high degree of dependence on third parties but also considerably increases the risk of falling. Our results found that patients who had suffered a stroke and who attended the DN and NRT session, exhibited greater improvements, as evidenced by the TUG test, increasing their stability when it came to changes in their centre of gravity.

This led us to believe that DN could favour the proprioception of the damaged hemibody, after normalizing the tone of the foot, allowing a considerable stability of the subtalar joint to be obtained, without forgetting that by normalizing the tone of the tibial musculature, results were observed at the tibioperoneal level (distal–proximal); this increased control in the face of imbalances and evidenced an increase in the ankle strategy, triggering a normalized ascending pattern of motor recruitment starting from the ankle, passing through the thigh muscles and ending at the hip, when normally patients, due to a lack of ankle stability, recruit from proximal to distal for postural control [27].

Similarly, Salom-Moreno et al. [28] conducted a clinical trial in which 27 patients with chronic unilateral ankle instability were treated with a combined programme of muscle strengthening, proprioception and DN on the lateral peroneus longus muscle. The results of this study suggested that the mechanical changes at the distal level in the stroke patient after dry needling indicated the activation of central mechanisms, especially descending inhibitory systems. The fact that a unilateral application of dry needling on the spastic leg musculature produced bilateral sensory effects at metameric levels unrelated to the treated region supported this theory whereby the descending inhibitory sensory systems were activated by central mechanisms.

It appears, therefore, that DN represents a suitable stimulus that activates the bilateral effects in distal areas (leg, foot), as obtained in our study. Such bilateral effects of DN could be mediated by different spinal reflexes, which depend on the afferent pathways from the remote site of stimulation to the spinal cord and on the normal function of the spinal dorsal horn at the level corresponding to the innervations of the affected proximal muscle. Audette et al. [29] observed that the unilateral puncture of a deep spinal muscle, in this case, the multifidus muscle, induced bilateral responses in the same spinal cord segment, confirming contralateral responses to unilateral stimulation.

Finally, these results were very similar to those of the study by Sánchez-Mila et al. [30], who concluded that both the control group treated with NRT and the intervention group treated with NRT plus a DN session obtained improvements in spasticity; however, the intervention group achieved a much greater improvement than the control group, which may have been due to the fact that NRT has very little effectiveness in a single treatment session in patients who have suffered a stroke. Therefore, despite being one of the most widely used therapies [31], the greater improvement of patients in the experimental group could clearly have been attributed to the introduction of DN. The changes that occurred in patients after the intervention could be useful for patients to advance in their particular day to day against their disability, since it is a technique that requires little time for its application, and it can enhance subsequent treatments, since the reduction in spasticity and the positional improvement of the patient invite to think that the subsequent work methods could be more effective.

Although our study only applied a single DN session on the leg musculature, the application of this tool could be performed on any part of the body as long as the anatomical considerations of each area are respected. Thus, Fakhari et al. [32] applied dry needling on the wrist flexors, analysing the improvement in decreasing wrist flexor spasticity and normalizing alpha motor neuron excitability.

### Limitations

Although the results of our clinical trial appear promising, potential limitations should be recognized and taken into account when interpreting the results. The first was the follow-up period. When performing the technique and its immediate evaluation, we could not know how long the effect of dry needling would have lasted, a very important aspect to consider. In future studies, it would be advisable to perform some type of longer-term follow-up to see if the improvements are maintained over time or if the patient returns to his or her baseline situation. This aspect is fundamental in the case of patients suffering from stroke, since in this case, unlike in the treatment of pain, the dry needling technique is always palliative.

## 5. Conclusions

It was demonstrated in a randomised, blind-controlled study that the inclusion of ultrasound-guided DN in a neuro-rehabilitation treatment session favoured a decrease in spasticity in the AT and PT, as well as an improvement in the risk of falls as measured with the use of TUG, with the aim of allowing patients to achieve an improvement in their quality of life by including the invasive technique of dry needling in the physiotherapy session.

## Figures and Tables

**Table 1:** Baseline clinical, demographics and functional scales.

	Group NRT	Group Dry Needling + NRT	*p*-Value
Gender (male/female)	6/4	7/3	
Age (mean ± sd)	52.4 ± 7.86	46.10 ± 7.78	=0.088
Affected side (right/left)	0/10	0/10	

**Table 2:** Changes in MMAS before and after intervention in both groups.

	Group NRT	Group Dry Needling + NRT	*p*-Value
MMAS tibial posterior pre-intervention n (%)			<0.001
Grade 2	5 (50%)	5 (50%)	
Grade 3	5 (50%)	5 (50%)	
MMAS tibial posterior post-intervention n (%)			
Grade 0	-	2 (50%)	
Grade 1	-	8 (80%)	
Grade 2	8 (80%)	-	
Grade 3	2 (20%)	-	
MMAS tibial anterior pre-intervention n (%)			<0.001
Grade 1	1 (10%)	1 (10%)	
Grade 2	5 (50%)	7 (70%)	
Grade 3	4 (40%)	2 (20%)	
MMAS tibial anterior post-intervention n (%)			
Grade 0	-	6 (60%)	
Grade 1	3 (30%)	4 (40%)	
Grade 2	6 (60%)	2 (20%)	
Grade 3	1 (10%)	-	

## Data Availability

The data presented in this study are available upon request from the corresponding author. The data are not publicly available due to compliance with data protection regulations.
